# TBC1D9: An Important Modulator of Tumorigenesis in Breast Cancer

**DOI:** 10.3390/cancers13143557

**Published:** 2021-07-16

**Authors:** Charu Kothari, Alisson Clemenceau, Geneviève Ouellette, Kaoutar Ennour-Idrissi, Annick Michaud, René C.-Gaudreault, Caroline Diorio, Francine Durocher

**Affiliations:** 1 Département de Médecine Moléculaire, Faculté de Médecine, Université Laval, Québec City, QC G1T 1C2, Canada; charu.kothari.1@ulaval.ca (C.K.); alisson.clemenceau.1@ulaval.ca (A.C.); genevieve.ouellette@crchudequebec.ulaval.ca (G.O.); Rene.C-Gaudreault@crchudequebec.ulaval.ca (R.C.-G.); 2 Centre de Recherche sur le Cancer, Centre de Recherche du CHU de Québec-Université Laval, Québec City, QC G1V 4G2, Canada; kaoutar.ennour-idrissi.1@ulaval.ca (K.E.-I.); Annick.Michaud@crchudequebec.ulaval.ca (A.M.); caroline.diorio@crchudequebec.ulaval.ca (C.D.); 3 Département de Biologie Moléculaire, de Biochimie Médicale et de Pathologie, Faculté de Médecine, Université Laval, Québec City, QC G1T 1C2, Canada; 4 Département de Médecine Sociale et Préventive, Faculté de Médecine, Université Laval, Québec City, QC G1T 1C2, Canada; 5 Laboratoire de Chimie Médicinale, l’Hôpital Saint-François d’Assise, Université Laval, Québec City, QC G1L 3L5, Canada; 6 Centre des Maladies du Sein, Hôpital du Saint-Sacrement, Québec City, QC G1S 4L8, Canada

**Keywords:** breast cancer, migration, tumorigenesis, TNBC, predictive marker, molecular subtypes, early detection

## Abstract

**Simple Summary:**

Triple negative breast cancer (TNBC) lacks the expression of hormonal receptors estrogen and progesterone along with the over-expression of human epithelial growth factor receptor 2 (HER2). The lack of these receptors makes the treatment targeting these receptors ineffective. TNBC is classified as the most aggressive BC with heterogeneity among patients, resulting in the lack of an effective treatment that could be used in all TNBC patients. This paper describes the result where we identified a low expression of *TBC1D9* gene in all TNBC patients (irrespective of their heterogeneity) as compared to non-TNBCs. Down-regulation of the expression of TBC1D9 in luminal BC and TNBC cell lines results in acquisition of more aggressive phenotype. This might link low expression of TBC1D9 in TNBC with its aggressive nature. These data suggest that modulation of TBC1D9 expression or its effector genes in TNBC patients could provide a new therapeutic hope for all TNBCs.

**Abstract:**

Triple-negative breast cancer (TNBC) is a major concern among the different subtypes of breast cancer (BC) due to the lack of effective treatment. In a previous study by our group aimed at understanding the difference between TNBC and non-TNBC tumors, we identified the gene TBC1 domain family member 9 (*TBC1D9*), the expression of which was lower in TNBC as compared to non-TNBC tumors. In the present study, analysis of TBC1D9 expression in TNBC (*n* = 58) and non-TNBC (*n* = 25) patient tumor samples validated that TBC1D9 expression can differentiate TNBC (low) from non-TNBC (high) samples and that expression of TBC1D9 was inversely correlated with grade and proliferative index. Moreover, we found that downregulation of the *TBC1D9* gene decreases the proliferation marginally in non-TNBC and was associated with increased migratory and tumorigenic potential in both TNBC and luminal BC cell lines. This increase was mediated by the upregulation of *ARL8A*, *ARL8B*, *PLK1*, *HIF1α*, *STAT3*, and *SPP1* expression in TBC1D9 knockdown cells. Our results suggest that TBC1D9 expression might limit tumor aggressiveness and that it has a differential expression in TNBC vs. non-TNBC tumors.

## 1. Introduction

Breast cancer (BC) is the second leading cause of cancer-associated death in women. Around 81% of BCs diagnosed in the United States are invasive and if diagnosed early the five-year survival rate is 91% (American Cancer Society 2020) [1]. However, only 62% of women with BC are diagnosed at stage 1, and a large population-based study suggests that the later the stage of the disease at the time of diagnosis, the poorer the survival outcome will be [2]. This issue is augmented with triple-negative breast cancer (TNBC), which is characterized by the lack of expression of the estrogen receptor (ER) and progesterone receptor (PR), and lack of the overexpression of the epidermal growth factor receptor 2 (HER2). TNBC accounts for 15–20% of all BC and is usually diagnosed at a later stage [3]. It is a heterogeneous and aggressive disease with a very high proliferative and metastatic potential [4]. It is not responsive to hormonal and targeted therapy (anti-HER2) commonly used to treat BC [5]. Furthermore, the molecular diversity within TNBC makes it even harder to treat [6]. The heterogeneity of TNBC is a major hurdle in the treatment of TNBC. According to the study by Lehmann et. al. in 2011, TNBC can be grouped into six molecular subtypes based on the cluster analysis performed on the gene expression pattern, and each of these subtypes can be further grouped into the intrinsic molecular subtypes of BC (basal-like, luminal A, luminal B, HER2, normal-like). This scenario makes it difficult to identify a therapeutic option that could be effective against all TNBCs. Therefore, it is essential to identify new gene targets that are differentially regulated in all TNBCs as compared to non-TNBCs, irrespective of the endogenous heterogeneity of TNBC.

A previous study by our group highlighted the *TBC1D9* gene as downregulated in TNBC as compared to non-TNBC tissue samples [7]. TBC1 domain family member 9 (TBC1D9) acts as a calcium-binding and GTPase-activating protein [8]. The expression of a combination of genes involving *TBC1D9* is inversely associated with disease mortality and relapse in BC [9]. In addition, the dysregulation of *TBC1D9* expression in BC is associated with cell proliferation disorder that leads to a non-proliferative fibrocystic change of the breast (MalaCards, Human Disease Database), a benign condition of the breast comprising fibroadenoma, cysts, fibrosis, and microcalcifications. Furthermore, the *TBC1D9* gene, along with a group of genes (*AGR3*, *CA12*, *AGR2*, *GATA3*, *FOXA1*, and *MLPH*), has been identified to be co-expressed with estrogen receptor *ESR1* in BC [10].

There is, however, a lack of data regarding the expression and function of TBC1D9 in general. In our previous work, a protein–protein interaction study of TBC1D9 showed that it regulates the mitotic and migratory potential of a cell by regulating ARL8A and PLK1 [7].

In the present study, we confirmed that the low expression of TBC1D9 is associated with TNBC subtypes and with high-grade BC. We also observed that the expression of TBC1D9 was higher in non-invasive BC (DCIS) as compared to invasive BC (IDC). Moreover, we observed a marginal decrease in the proliferation of non-TNBC cell lines upon the downregulation of *TBC1D9*. However, an increase in migratory and tumorigenic potential was observed upon knockdown (KD) of *TBC1D9* in both non-TNBC and TNBC cell lines by regulating the expression of *ARL8A*, *PLK1*, *HIF1α*, *STAT3*, and *SPP1*. The present study sheds light on the role of TBC1D9 as a modulator of BC aggressiveness and underlines the fact that the aggressiveness of TNBC could be due to the lack of expression of TBC1D9. To date, there is no FDA-approved target for TNBC subtypes, except for immunotherapy with Atezolizumab (targeting PD-L1), which is approved for women with TNBC expressing the PD-L1 protein [11]. Around 41% of TNBCs are PDL1-positive, of which only 59% show a positive response to this combination [12]. Hence, the low expression of TBC1D9 across different TNBC molecular subtypes could be an asset. Indeed, modulating the expression of *TBC1D9* or the genes affected by the expression of TBC1D9, either alone or in combination with other available therapeutic options, could provide new hope to women diagnosed with TNBC, as the expression of *TBC1D9* is downregulated in all TNBCs despite its heterogeneity. Furthermore, following the expression pattern of *TBC1D9* in clinics could predict which BC could become invasive, as the decrease in *TBC1D9* increases the tumorigenicity of BC.

## 2. Materials and Methods

### 2.1. Public BC Datasets Analysis

The difference in *TBC1D9* expression pattern between the normal breast tissue and tumoral tissue samples, as well as with different molecular subtypes of BC (luminal A, luminal B, HER2, and TNBC), was analyzed using data from the gene expression database of normal and tumor tissues (http://gent2.appex.kr/gent2/; accessed on 11 January 2021) [13]. The statistical analysis of significance was done using GraphPad Prism 8. The protein expression of TBC1D9 in different subtypes of BC was analyzed in the UALCAN database (http://ualcan.path.uab.edu/ accessed on 11 January 2021) using data from the Clinical Proteomic Tumor Analysis Consortium (CPTAC) analysis [14]. For normal breast vs. BC tissue samples, an unpaired Student’s *t*-test was used, whereas Kruskal–Wallis one-way analysis of variance was used to determine the significance of the differences in the expression pattern of *TBC1D9* in different BC subtypes. The term “basal” was used for the set of tissue samples with basal characteristics but which could not be classified as TNBC due to the presence of ER/PR/HER2 expression.

Furthermore, the effect of *TBC1D9* expression on survival was analyzed using the Kaplan–Meier plotter (https://kmplot.com/analysis/, accessed on 7 June 2021) [15]. Relapse-free survival (RFS; *n* = 3951), and overall survival (OS; *n* = 1402) were analyzed using the median expression of *TBC1D9* as a cutoff.

### 2.2. Breast Tissue Samples Selection and Tissue Microarray (TMA) Preparation

The breast lesion tissue samples were provided by the tissue bank located at the Centre des Maladies du Sein of Hôpital du St-Sacrement, Quebec, Canada. Breast tissue samples (*n* = 83) deposited in the biobank between 2010 to 2014 from women (mean age: 56.0 ± 13.9 years) with no hormonotherapy or chemotherapy treatment before surgery were selected for this study, consisting of TNBC (*n* = 58), non-TNBC (*n* = 12), and ductal carcinoma in situ (DCIS; *n* = 13). All DCIS were ER-positive. All breast diseases were confirmed by an experienced breast pathologist, and all tumor characteristics were routinely collected from medical reports: tumor size, histologic type, grade, lymph node involvement, and receptor status (ER, PR, and HER2). Clinical information on BC patients was obtained from their medical reports. Formalin-fixed paraffin-embedded (FFPE) blocks containing tumoral tissue were selected by a pathologist specialized in breast pathology.

Classification of TNBC and non-TNBC subtypes was based on the expression pattern of the hormonal receptors ER and PR and the HER2 receptor. In brief, luminal A is ER- and/or PR-positive and HER2-negative; luminal B is ER- and/or PR-positive and HER2-positive: HER2 is ER- and PR-negative and HER2-positive; TNBC is ER-, PR-, and HER2-negative. All DCISs were ER-positive.

TMAs were constructed from the FFPE mastectomy blocks containing BC tissue samples as previously described [16].

### 2.3. Immunohistochemistry (IHC) Analysis and Scoring

The IHC staining was performed as previously described [17] with some modifications. For TBC1D9 staining, 3% H_2_O_2_ was used to block the endogenous peroxidase, non-specific binding was blocked for 1 h at room temperature (RT) using Superblock (IDetect™ Super Stain System HRP), and the staining was done by incubating the slides for 2 min at RT with chromogen substrate 3,3′-diaminobenzidine (DAB; Empire Genomics, Buffalo, NY, USA). For Claudin 3 and cluster of differentiation 24 (CD24), antigen retrieval was done using Tris-EDTA (pH 9) and a mouse-specific secondary antibody (catalog number: K4001, Dako EnVision^+^ system HRP) was used. The primary antibody against TBC1D9 was used with a 1:600 dilution (anti-rabbit, catalog number: HPA000262, Sigma-Aldrich, Germany). The primary antibodies against Claudin 3 (anti-mouse; catalog number: (1E7)-sc-517546), Claudin 4 (anti-mouse; catalog number: (A-12)-sc-376643), and CD24 (anti-mouse; catalog number: (M1/69)-sc-19651) were used with 1:50 dilutions and were purchased from Santa Cruz Biotechnology, Inc., Texas, United States. Ki-67 staining was performed at the clinical pathology platform at Hôpital du St-Sacrement, Quebec, Canada. A representative image of IHC staining is presented in Figure S1.

Expression of Ki-67 was analyzed in all BC tissue samples to evaluate their proliferative index. For molecular characterization of TNBC samples, expressions of CD24, Claudin 3, Claudin 4, and androgen receptor (AR) were analyzed in our cohort according to Lehmann et al., 2011 [6]. Tissue samples expressing more than 70% Ki-67 staining were considered basal-like (BL). Low Ki-67 staining (<70%) accompanied by low Claudin (both Claudin 3 and 4) and CD24 staining was considered mesenchymal-like (M). The median H-score was taken as a cutoff for classifying Claudin 3 (low = H-score < 100), Claudin 4 (low = H-score < 95), and CD24 (low = H-score < 160) as high or low. TNBC tissue samples expressing more than 10% AR nuclear staining were considered positive and were characterized as luminal androgen receptor (LAR) TNBC [18]. The information regarding the immune-modulatory (IM) TNBC was extracted from the histo-pathological clinical report, identifying the tumor as medullary carcinoma.

The analysis of TBC1D9, Claudin 3, Claudin 4, and CD24 staining was done by analyzing the percentage of cancer cells stained, the intensity of staining, and the heterogeneity of staining intensities. Scoring for intensity corresponds to 0 = no staining; 1 = low intensity; 2 = medium intensity; and 3 = high intensity. The H-score was calculated according to the percentage of cells stained and the intensity of staining [19]. Ki-67 staining analysis was done as previously described [20]. Reproducibility of the scoring for TBC1D9 was examined by independent scoring of 10% of the cores randomly selected from the TMAs by a pathologist (r > 0.6).

### 2.4. Cell Lines

For TBC1D9 analysis, we used five non-TNBC cell lines, namely MCF7, T47D, BT474, SKBr3, and JIMT-1, whereas three TNBC cell lines were used, MDA-MB-231, SUM159PT, and SUM149PT. Two additional TNBC cell lines were used to evaluate the efficacy of TNBC cell lines in a chick chorioallantoic membrane (CAM) assay, namely MDA-MB-468 and BT-549. The molecular classification of these cell lines is indicated in Table S1. SKBr3 was cultured in McCoy media. For the two SUM cell lines, Ham’s F-12 culture media containing 5% fetal bovine serum (FBS), 1 µg/mL hydrocortisone, 10 mM HEPES, and 5 µg/mL insulin was used. RPMI-1640 was used for MDA-MB-231, MDA-MB-468, and BT-549. BT-549 media was supplemented with 0.002 mg/mL insulin. DMEM-F12 supplemented with 5% FBS, 10 mM HEPES, 0.2% sodium bicarbonate, and 10 nM estradiol (E2) was used for MCF7. T47D was grown in RPMI-1640 (without phenol red) supplemented with 1% glutamine, 10 mM HEPES, 0.2% sodium bicarbonate, and 10 nM E2. DMEM supplemented with 2 mM glutamine was used for JIMT-1 cells. All media were supplemented with 1% penicillin-streptomycin mixture (5000 IU penicillin, 5000 μg/mL) and 10% FBS, except where otherwise indicated. All cell culture reagents were purchased from Wisent Inc. (Quebec, QC, Canada).

### 2.5. RNA Extraction and Quantitative Real-Time PCR (qPCR) Analysis

Total RNA from breast tissue samples and BC cell lines were respectively isolated using a Qiagen RNeasy FFPE Kit and Qiagen RNeasy Mini Kit (Qiagen, Hilden, Germany).

Quantitative PCR was performed using SYBR Green technology as described previously [21,22]. The sequence of primers is indicated in Table S2. Data calculation and normalization were performed using the second-derivative and double-correction method [23], with two housekeeping (HK) genes (*HPRT1* and *GAPDH*). For signaling analysis, *GAPDH* was used as the HK gene. The mRNA levels are indicated as quantities relative to HK genes.

### 2.6. Lentiviral Production and Infection

Two lentiviruses (TRCN0000055711 and TRCN0000055712) against *TBC1D9* were analyzed. Throughout the article, we have used the names sh*TBC1D9*-711 and sh*TBC1D9*-712 for TRCN0000055711 and TRCN0000055712 respectively. The shRNAs were a kind gift from Prof. S. Gobeil, Université Laval, Quebec, Canada. Lentivirus packaging and virus infection were done as previously described [24]. Briefly, lentiviral constructs with scrambled shRNA and *TBC1D9* shRNA were transfected in HEK293T cell lines with the lentiviral packaging vectors. The transfection media were changed after 12–16 h of transfection with a complete medium for the HEK293T cell lines and the virus-containing media were collected after 48 h. The virus-containing media were filtered and BC cell lines were transfected with the 10% virus-containing medium with 10 µg/mL polybrene. The cells infected with shScrambled are labeled as shControl throughout the article.

### 2.7. Proliferation

The control BC cell lines (shControl) and BC cell lines with *TBC1D9* knockdown (KD), were seeded in 96 well microtitre plates (10,000 cells/well). After 72 h of incubation at 37 °C with 5% CO_2_, a proliferation assay was performed using Alamar blue (ThemoFisher Scientific, Waltham, Ma, USA) by following the manufacturer’s protocol. The difference in proliferation was detected by measuring the fluorescence using an excitation between 530–560 nm and emission at 590 nm with an Infinite M1000 Pro plate reader (Tecan Austria GmbH, Grödig, Austria). Graphs are representative of three biological replicates.

### 2.8. Migration Assay

A wound-healing assay was performed according to the manufacturer’s protocol (iBidi, Martinsried, Planegg, Germany). Briefly, cells were trypsinized and 100 µL of culture media containing 25,000 cells (shControl and *TBC1D9* KD) was added in each well of the two-well silicone insert. The plates containing the insert were incubated at 37 °C with 5% CO_2_ for 16 h. The insert was removed and the cells were washed twice with 1X phosphate-buffered saline and allowed to migrate after the addition of media with 10 µM mitomycin C. The images were taken at the indicated time with an EVOS™ M5000 Imaging System (Invitrogen™, ThermoFisher Scientific, Waltham, MA, USA). Images are representative of two biological replicates.

### 2.9. Three-Dimensional Spheroid Formation Assay

BC cells (control and *TBC1D9* KD) were trypsinized and 5000 cells were suspended in complete media and seeded (5000 cells/well) in Corning^®^ 96-well clear, round-bottom, ultra-low-attachment microplates (Millipore Sigma, Oakville, ON, Canada). The cells were incubated at 37 °C with 5% CO_2_ for 7 days. The images were taken with an EVOS™ M5000 Imaging System (Invitrogen™, ThermoFisher Scientific, Waltham, MA, USA). Images are representative of two biological replicates.

### 2.10. CAM Assay

The assay was performed according to the published protocol [25]. Briefly, fertilized chicken eggs were purchased and incubated at 37 °C with 60% humidity. On the eleventh day of embryo development, a small window (CAM site) was made in the shell under aseptic conditions and the BC cell suspension (shControl and sh*TBC1D9*) was added at the CAM site, resealed with adhesive tape, and returned to the incubator. Seven days after the addition of cells, the eggs were sacrificed, tumours were removed, and images were taken. Ten eggs were used for each group (shControl, sh*TBC1D9*-711, and sh*TBC1D9*-712). The representative images are from two independent biological experiments.

### 2.11. Statistical Analysis

All IHC analyses were performed with GraphPad Prism8 (GraphPad Software, San Diego, CA, USA), and some data were also correlated using RStudio v1.2.5033 (RStudio Team (2019), RStudio: Integrated Development for R, RStudio, Inc. (Boston, MA, USA)). The Spearman correlation analysis was performed to correlate the RNA and protein expression of TBC1D9 in BC tissue samples from our cohort. Correlation of continuous variables with TBC1D9 expression in BC tissue samples from our cohort was also done using the Spearman correlation. *p*-values lower than 0.05 were considered significant. The Wilcoxon–Mann–Whitney test was used to evaluate the significance of the differences observed in the in vitro and in vivo assays.

## 3. Results

### 3.1. Low TBC1D9 Expression Correlates with Poor Survival Outcomes

**Figure 1:**
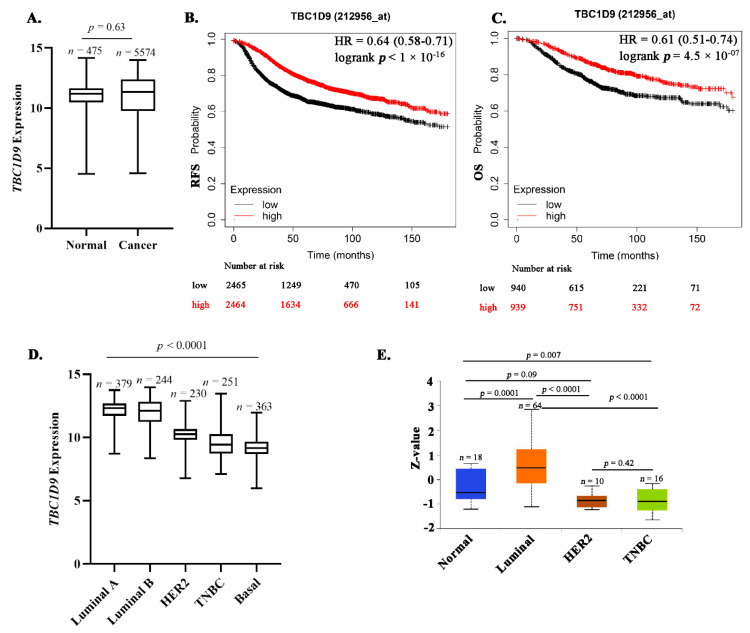
Expression of *TBC1D9* in publicly available BC datasets and its effect on survival. *TBC1D9* expression in normal (*n* = 475) vs. BC (*n* = 5574) tissue samples (GENT2 database; http://gent2.appex.kr/gent2/, accessed on 11 January 2021) (**A**). Effect of *TBC1D9* on the survival outcome of BC patients: RFS (**B**) and OS (**C**) (https://kmplot.com/analysis/, accessed on 7 June 2021). HR = hazard ratio. TBC1D9 expression in different subtypes of BC: mRNA (GENT2 database) (**D**) and protein (UALCAN database; http://ualcan.path.uab.edu/, accessed on 11 January 2021) (**E**).

To better understand *TBC1D9* expression in BC, we compared its expression in normal (*n* = 475) and BC (*n* = 5574) tissue samples from the GENT2 database and did not find any significant difference (*p* = 0.63, Figure 1A). We further analyzed the effect of *TBC1D9* expression on survival outcome of BC patients and found that the low expression of *TBC1D9* correlates with poor survival outcome for RFS (*p* = 1.5 × 10^−^^13^, Figure 1B) and OS (*p* = 1.5 × 10^−^^5^, Figure 1C). We then compared its expression in different subtypes of BC from an online BC dataset (GENT2) and found that its expression was lowest in the TNBC or basal subgroup of BC (*p* < 0.0001; Figure 1D). Similar results were obtained when we compared the protein expression pattern of TBC1D9 using a different online BC dataset (UALCAN) consisting of normal (*n* = 18), luminal (*n* = 64), HER2 (*n* = 10), and triple-negative (*n* = 16) breast cancers, except for HER2, where TBC1D9 expression was similar to TNBC (Figure 1E).

### 3.2. Confirmation That TBC1D9 Can Differentiate TNBC from Other BCs

**Figure 2:**
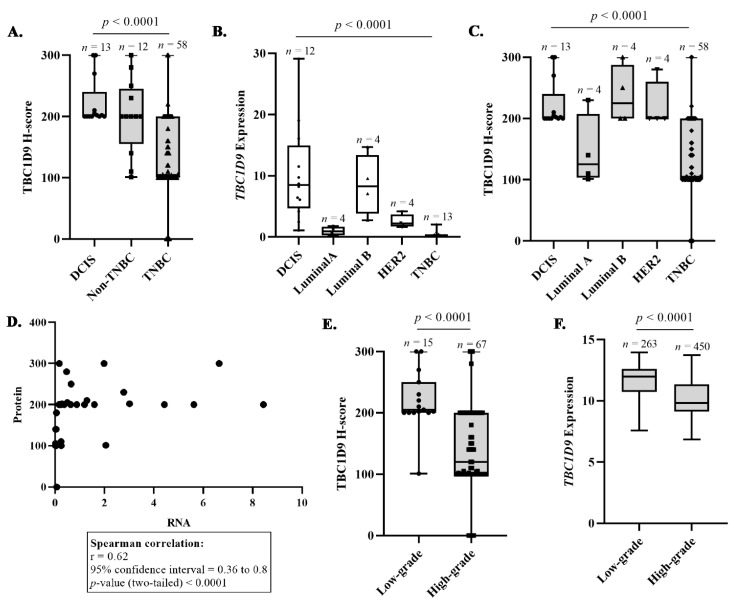
Analysis of TBC1D9 expression in BC cohorts. The differences in the expression patterns of TBC1D9 in DCIS, non-TNBC, and TNBC tissue samples in our cohort (**A**). Expression of *TBC1D9* in different subtypes of BC: DCIS (*n* = 12), luminal A (*n* = 4), luminal B (*n* = 4), HER2 (*n* = 4), and TNBC (*n* = 13) (**B**). Expression of TBC1D9 in different subtypes of BC: DCIS (*n* = 13), luminal A (*n* = 4), luminal B (*n* = 4), HER2 (*n* = 4), and TNBC (*n* = 58) (**C**). Spearman correlation between mRNA and protein expression of TBC1D9 in BC tissue samples from our cohort (**D**). Expression pattern of TBC1D9 in BC tissue samples according to grade in our cohort (**E**) and a cohort from the online database GENT2 (**F**).

Protein and RNA expressions of TBC1D9 were assessed in 83 and 37 BC tissue samples, respectively, from our cohort. The Spearman correlation between RNA and protein expressions of TBC1D9 in BC samples from our cohort was high (rho = 0.62, *p* < 0.0001, Figure 2D). We observed that the expression of TBC1D9 was lower in IDC as compared to DCIS BC tissues in our cohort (Figure S2A, *p* < 0.0001). To verify our finding that TBC1D9 expression can differentiate TNBC from non-TNBC, we stratified our cohort as DCIS, non-TNBC, and TNBC, and we observed that the expression of TBC1D9 was significantly lower in TNBC as compared to DCIS and non-TNBC samples (*p* < 0.0001, Figure 2A). Similar results were observed at RNA levels (data not shown). High levels of TBC1D9 were also associated with the stage of the disease in all BCs (*p* = 0.048, Figure S2B). Next, we stratified our cohort according to subtypes of BC and observed that TBC1D9 expression was lower in TNBC as compared to other BC subtypes, except for luminal A, both at the RNA (*p* < 0.0001, Figure 2B) and protein (*p* < 0.0001, Figure 2C) levels. Furthermore, we stratified our TNBC subgroups according to their molecular subtypes (BL, IM, LAR, M, and UNS) and determined that expression of TBC1D9 was low in all TNBC compared to non-TNBC samples except for luminal A (*p* <0.0001, Figure S2C).

These data suggest that low expression of TBC1D9 correlates with the TNBC subgroup.

### 3.3. TBC1D9 Expression Inversely Correlates with Grade and Tumor Size

Next, we examined the expression of TBC1D9 by grades, where grade 1 (*n* = 1) and grade 2 (*n* = 14) of all BCs were grouped as low-grade tumors and were compared to high-grade tumors (grade 3, *n* = 67) for all BC tissue samples. We observed that low expression of TBC1D9 was associated with the high-grade tumor (*p* < 0.0001; Figure 2E). A similar pattern was observed when we analyzed IDC separately, with a *p*-value of 0.002 (Figure S2D), but not for the DCIS group (Figure S2E). The analysis of the BC dataset from the GENT2 database showed a similar difference in the TBC1D9 RNA expression in different BC tumor grades (Figure 2F).

Furthermore, to better understand the involvement of TBC1D9 in BC, we performed Spearman correlations between TBC1D9 expression and different tumoral parameters. The analysis was performed by stratifying the BC tissue samples in different groups: all BCs, IDC, TNBC, non-TNBC including DCIS, and non-TNBC. The parameters analyzed were the size of the tumor, the proliferation index (Ki-67 expression), CD24, Claudin 3, and Claudin 4 expression. Tumor size is the basis of the American Joint Committee on Cancer’s (AJCC) cancer staging principles and is the most frequently used prognosis parameter for BC [26]. We found that the low expression of TBC1D9 was inversely correlated with tumor size in all BC tissue samples (rho = −0.25, *p* = 0.02) and in IDC (rho = −0.22, *p* = 0.06), non-TNBC including DCIS (rho = −0.49, *p* = 0.01), and non-TNBC (rho = −0.80, *p* = 0.01) tissue samples, but not in TNBC (rho = −0.14, *p* = 0.29) (Table 1). High and uncontrolled proliferation is one of the prominent characteristics of malignancy and can be assessed by Ki-67 staining [27]. In the present study, we found that the TBC1D9 expression was inversely correlated with Ki-67 expression when all BC tissue samples were considered (rho = −0.29, *p* = 0.008) (Table 1).

Claudin 3, Claudin 4, and CD24 were used in the study to classify TNBCs into their molecular subtypes. However, we analyzed the correlation of TBC1D9 expression with Claudin 3, Claudin 4, and CD24 expression in all BCs, as they all play a role in increased breast tumorigenesis [28,29,30,31,32,33]. Our data showed that a high expression of TBC1D9 was positively correlated with CD24 expression in all BC tissue samples (rho = 0.20, *p* = 0.07) and in IDC (rho = 0.27, *p* = 0.02) and TNBC samples (rho = 0.36, *p* = 0.006) but not in non-TNBC samples including DCIS (rho = −0.09, *p* = 0.66) or without DCIS (rho = −0.09, *p* = 0.85) (Table 1). We did not observe a significant correlation of TBC1D9 with Claudin 3 and Claudin 4.

These data suggest that low TBC1D9 expression inversely correlates with tumor grade and size. Moreover, expression of TBC1D9 positively correlated with CD24 expression in the IDC and TNBC subgroups and negatively correlated with proliferation rate when analyzed in all BC tissue samples.

### 3.4. TBC1D9 KD Increases Cell Migration and 3D Spheroid Formation in Luminal and TNBC Cell Lines

**Figure 3:**
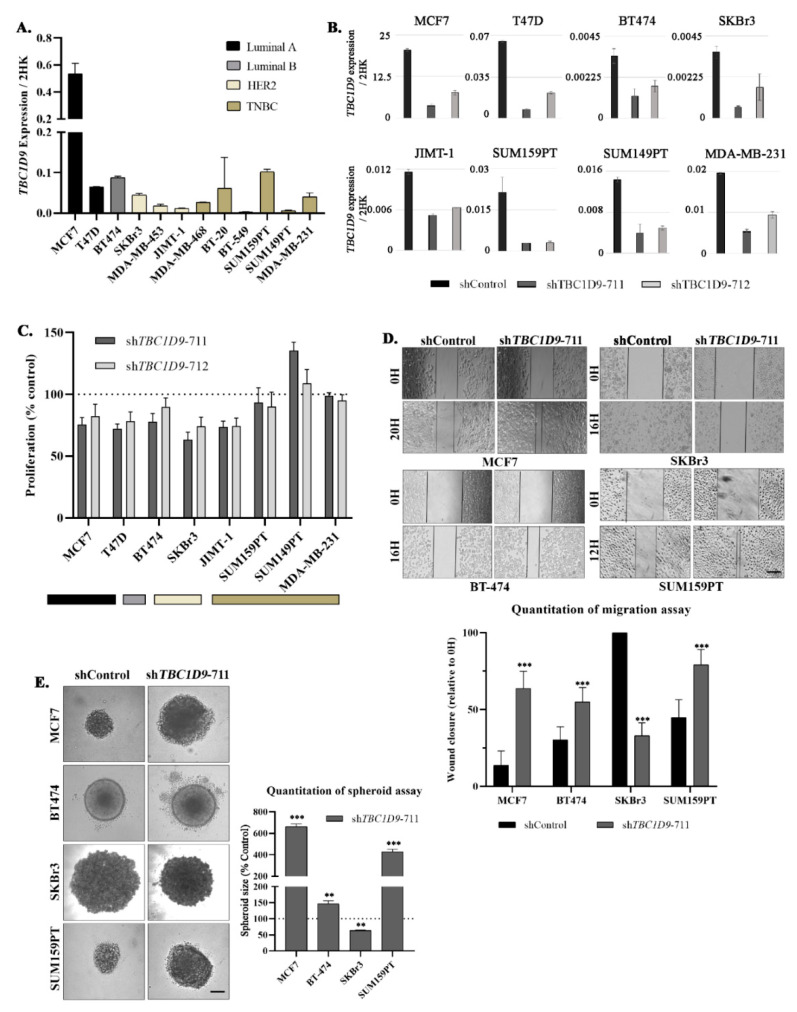
Effect of *TBC1D9* knockdown on different phenotypes of BC. Screening of *TBC1D9* expression in different BC cell lines (**A**). Validation of *TBC1D9* knockdown by sh*TBC1D9*-711 and sh*TBC1D9*-712 in BC cell lines: luminal A (MCF7, T47D), luminal B (BT474), HER2 (SKBr3, JiMT-1), and TNBC (SUM159PT, SUM149PT, and MDA-MB-468), with the two housekeeping genes (2HK) *GAPDH* and *HPRT1* (**B**). Effect of *TBC1D9* knockdown on proliferation (**C**), migration (**D**), and the 3D spheroid formation assay (**E**). Scale bar: 100 µm. H = hour; ** *p* = 0.005; *** *p* = 0.0005.

To understand the role of the *TBC1D9* gene in BC we performed qPCR to analyze the expression pattern of *TBC1D9* in different BC cell lines to best select the cell lines for our in vitro and in vivo experiments (Figure 3A). As we did not see a particular trend in the expression pattern of *TBC1D9* in the cell lines, we chose two luminal A (MCF7, T47D), one luminal B (BT-474), two HER2 (SKBr3, JIMT-1), and three TNBC (SUM159PT, SUM149PT, and MDA-MB-231) cell lines to test the efficacy of the two shRNAs (shTBC1D9-711 and shTBC1D9-712). Both shRNAs were effective in knocking down *TBC1D9*; however, shTBC1D9-711 had higher efficacy than shTBC1D9-712 in all instances (Figure 3B).

We then analyzed the effect of KD of *TBC1D9* with the two shRNA on the proliferation of the selected BC cell lines (Figure 3C). We observed a decrease of around 20–25% in luminal A (MCF7 and T47D) cell lines. Moreover, we observed a 25–36% decrease in SKBr3 (HER2), a decrease of around 25% in JIMT-1 (HER2), and a decrease of around 10–20% in BT-474 (luminal B). In TNBC cell lines, we observed either an increase or almost no change in proliferation (Figure 3C).

Next, we analyzed the effect of KD of *TBC1D9* on the migration of four BC cell lines (MCF7, BT-474, SKBr3, and SUM159PT). An increase in the migratory potential was observed for MCF7 (42.4%, *p* < 0.0001), BT-474 (24.8%, *p* = 0.0001), and SUM159PT (32.7%, *p* < 0.0001) and a decrease for SKBr3 (66.3%, *p* < 0.0001) upon *TBC1D9* KD as compared to control (Figure 3D). Moreover, an increase in the size of spheroids was observed with a 3D spheroid formation assay for MCF7 (6.6-fold, *p* < 0.0001), BT-474 (1.5-fold, *p* = 0.006), and SUM159PT (4.3-fold, *p* = 0.0001) and a 1.5-fold decrease was obtained in SKBr3 (*p* = 0.008) in *TBC1D9* KD cells as compared to control (Figure 3E).

These results indicate that TBC1D9 could be a suppressor of migration and tumorigenesis in luminal and TNBC cell lines and the opposite might be true for the HER2 cell line.

### 3.5. TBC1D9 KD Increases Tumor Growth of BC Cell Lines In Vivo

**Figure 4:**
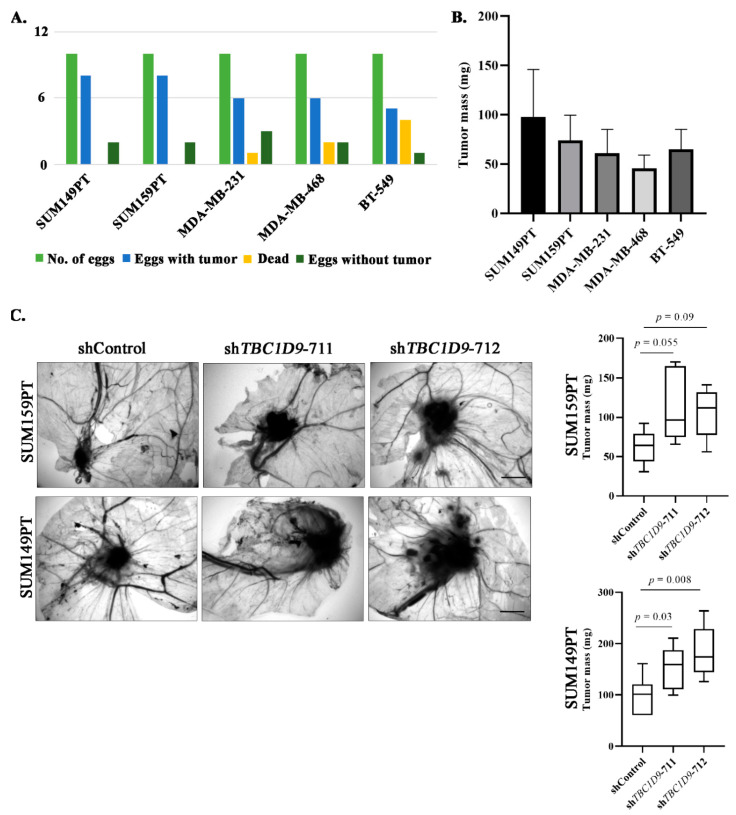
Effect of *TBC1D9* knockdown on BC tumor growth in an in vivo setting. Examination of different TNBC cell lines in a CAM assay to select the best model: outcomes of CAM assay (**A**) and average tumor mass (**B**). The effect of *TBC1D9* knockdown on tumor growth of SUM159PT and SUM149PT in the CAM assay (**C**). Scale bar: 100 µm.

As the 3D spheroid formation assay indicated that *TBC1D9* has an inhibitory effect on tumor growth (Figure 3E), this prompted us to further verify this finding in an in vivo system using a CAM assay. We first performed a CAM assay to choose which TNBC cell line had the best tumorigenic potential. We analyzed five different TNBC cell lines (SUM149PT, SUM159PT, MDA-MB-231, MDA-MB-468, and BT-549) in the CAM assay to assess their potential to form a tumor in the egg. Our initial experiment showed the best results with SUM-159PT and SUM149PT in terms of the numbers of eggs with tumor (Figure 4A) and tumor mass (Figure 4B). We then performed another CAM assay in these two cell lines with shControl, sh*TBC1D9*-711, and sh*TBC1D9*-712. The results of the CAM assay were comparable to those obtained with the 3D spheroid formation assay. Indeed, we observed increases in tumor size of 1.9-fold (*p*-value 0.055) and 1.7-fold (*p*-value 0.09) in eggs with SUM159PT cells with sh*TBC1D9*-711 and sh*TBC1D9*-712, respectively (Figure 4C), whereas increases of 1.6-fold (*p*-value 0.03) and 1.9-fold (*p*-value 0.008) were observed in eggs with SUM149PT cells with sh*TBC1D9*-711 and sh*TBC1D9*-712, respectively (Figure 4C). These data further confirmed our finding that *TBC1D9* acts as a tumor suppressor in luminal and TNBC cell lines. However, in HER2-positive cell lines, it acts as a tumor promoter.

### 3.6. The Effect of TBC1D9 on BC Phenotypes Is Mediated by Genes Involved in Tumor Growth and Migration

**Figure 5:**
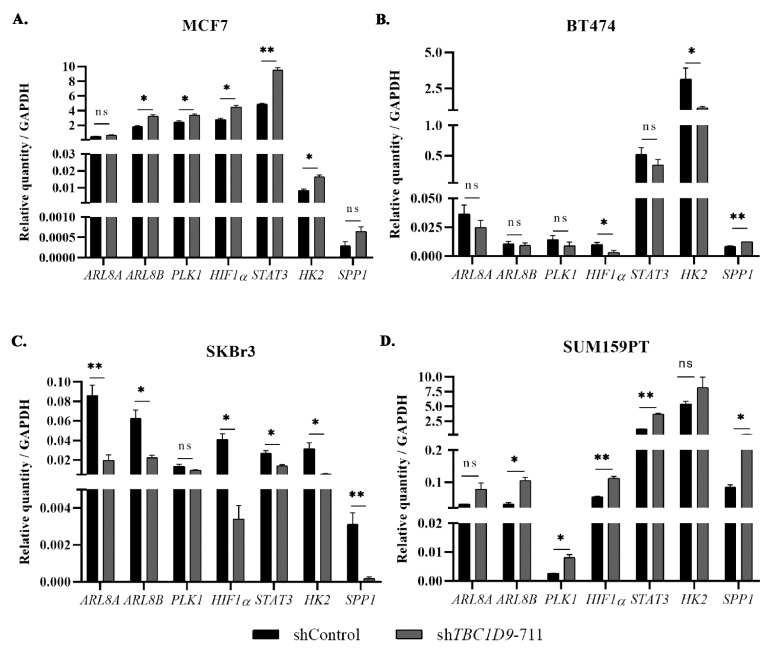
Signaling involved in cancer altered by *TBC1D9* knockdown in BC cell lines. Effect of *TBC1D9* knockdown on mRNA expression of *ARL8A*, *ARL8B*, *PLK1*, *SPP1*, *STAT3*, *HK2*, and *HIF-1α* in MCF7 (luminal A) (**A**), BT-474 (luminal B) (**B**), SKBr3 (HER2) (**C**), and SUM159PT (TNBC) (**D**). HK = *GAPDH*; * *p* = 0.05, ** *p* = 0.005; ns: not significant.

The interesting effect of *TBC1D9* on migration and tumor growth in luminal BC and TNBC led us to investigate the signaling involved in mediating these effects and how the signaling in BC with HER2 expression differs from other BC subtypes. In a previous study, we identified using affinity purification mass spectrometry (AP-MS) and proximity biotinylation (BioID) assays that TBC1D9 interacts with ARL8A and PLK1, and we hypothesized that, by restraining the expression of these genes, TBC1D9 might mediate its effect as a tumor suppressor by inhibiting cell division, migration, and extracellular matrix remodeling [7]. In the present study, we performed a qPCR analysis in BC cell lines with *TBC1D9* KD and analyzed genes responsible for these processes. The results indicated an increase in *ADP ribosylation factor-like GTPase 8A* (*ARL8A*), *ARL8B*, *polo-like kinase 1* (*PLK1*), *hypoxia-inducible factor 1 subunit alpha* (*HIF1α*), *hexokinase 2* (*HK2*), *secreted phosphoprotein 1* (*SPP1*), and *signal transducer and activator of transcription 3* (*STAT3*) in MCF7 (Figure 5A) and SUM159PT (Figure 5D). However, we observed the opposite in BT-474 (Figure 5B) and SKBr3 (Figure 5C). The only exception was *SPP1*, where an increase in the expression was observed in BT-474. This might explain the difference in phenotypic changes observed in HER2-positive cell lines. To confirm our findings, we evaluated these results in other luminal A (T47D), TNBC (SUM149PT), and HER2 (JIMT-1) cell lines (Figure S3) and observed similar results. For T47D and JIMT-1, *SPP1* was not detectable by qPCR analysis in our samples.

## 4. Discussion

BC remains a global health issue. With the advent of new diagnostic and treatment approaches, the mortality rate of BC has decreased in developed countries, but the incidence rate has increased significantly. Still, it has been estimated that 1 out of 33 women will die due to BC [1]. The heterogeneity of this disease is a major issue in identifying a potential therapy for BC [6]. However, understanding the histopathological and molecular differences in BC is crucial for proper management of the disease, as each patient responds differently to a given therapy. The problem becomes more prominent in TNBC, where hormonal therapy and targeted therapy against HER2 are ineffective due to the absence of ER, PR, and HER2 receptors [5]. Moreover, the variation within the TNBC subgroup makes it even more challenging, resulting in the present scenario where we do not have any effective target against TNBC which could be used for all TNBCs [5]. In a previous study by our group, we identified *TBC1D9* as downregulated in TNBC compared to non-TNBC [7]. The present study highlights that TBC1D9 has a differential expression in TNBC (low) and non-TNBC (high) tissue samples (Figure 2A) at the RNA and protein levels. Moreover, the low expression of TBC1D9 correlated with high-grade BC in our cohort (Figure 2E), which held true when restricted to IDC only (Figure S2D). As our cohort consisted of a large number of TNBC (*n* = 58) samples with a limited number of grade 2 (*n* = 2) samples, which might have influenced our results, we analyzed an online dataset to confirm our findings. This analysis revealed similar results, indicating that low expression of TBC1D9 indeed correlates with high-grade tumors (Figure 2F).

Furthermore, if we look at different subgroups of BC, the expression of TBC1D9 was lowest in the TNBC subgroup (Figure 2B,C) irrespective of TNBC heterogeneity (Figure S2C). Its low expression also correlated with worse survival outcomes (Figure 1D,E). This is concordant with Andres et al. (2013), who found that a high expression of TBC1D9 can predict a better survival outcome in BC patients when mortality and recurrence are analyzed [9]. Over decades, tumor size—a clinical component of the stage of BC—has been considered an important parameter in analyzing the survival outcome of BC patients [34]. The association of low expression of TBC1D9 with large tumor size observed in all BC patients may explain the positive correlation of the low expression of TBC1D9 with advanced stage BC in this population of patients. Additionally, our data also highlight that the expression of TBC1D9 negatively correlates with Ki-67 expression. Ki-67 expression is a marker of cell division and proliferation, and a study by Soliman et al. (2016) has shown that BC patients with high expression of Ki-67 (>15%) showed a higher risk of metastasis and recurrence [27], which is in line with our findings. Our data indicated that low expression of TBC1D9 positively correlated with CD24 expression specifically in TNBC (*p* = 0.006) (Table 1). It has been shown that the presence and absence of CD24 expression in TNBCs sensitize TNBCs to doxorubicin and docetaxel treatments, respectively, and could be used as a selective marker for the choice of chemotherapy in TNBC [35]. Based on this data showing an association of CD24 expression with the response to chemotherapy treatment, we could speculate that the presence of TBC1D9 could sensitize TNBC to doxorubicin treatment as well. Our data showed low expression of TBC1D9 in TNBC (as a group) as compared to other BCs but, if there is an expression of TBC1D9 in TNBC, it might be responsive to doxorubicin. However, very little is known about TBC1D9 in BC, and a thorough analysis is required for a better understanding.

The analysis of public BC datasets and our cohort confirmed our findings that the expression of TBC1D9 is indeed low in TNBC and correlates with worse survival outcomes (Figure 1 and Figure 3A). In line with these findings, KD of *TBC1D9* in BC cell lines showed an increase in the tumor size of BC in an in vivo setting using TNBC cell lines (Figure 4C) and an increase in migration (Figure 3D) and 3D spheroid formation (Figure 3E) in luminal and TNBC cell lines. Investigation of altered signaling due to *TBC1D9* KD led to the identification of *ARL8A*, *ARL8B*, *PLK1*, *SPP1*, *STAT3*, *HK2*, and *HIF-1α* as potential mediators of the phenotypic effects observed from *TBC1D9* KD. All these genes were upregulated in luminal A and TNBC cell lines. The opposite was true for luminal B and HER2 cell lines, except for the upregulation of *SPP1* in BT-474 (Figure 5, Figure S3). The difference could have been due to the overexpression of HER2, as the luminal B cell line in our study also overexpresses HER2. However, it seems that *TBC1D9* behaves differently in HER2-positive BC, where it might act as a tumor promoter in the absence of ER.

In our previous paper, we showed using affinity purification mass spectrometry and BioID experiments that TBC1D9 interacts with ARL8A and PLK1 [7]. In this study, we found that it downregulates both proteins (ARL8A and PLK1) through physical interaction. ARL8A is upregulated in many cancers [36,37], including inflammatory BC [38], and has been associated with poor survival outcomes. Moreover, PLK1 has been shown to play a role in TNBC progression, metastasis, and resistance [39,40,41]. A study by Wu et al. (2020) showed that lysosomal trafficking mediated by ARL8B promotes invasion of BC and is associated with poor survival outcomes [42], which was also confirmed by another study in both BC and prostate cancer [43].

We also observed an increase in *SPP1*, *STAT3*, *HK2*, and *HIF-1α* in *TBC1D9* KD luminal A and TNBC cell lines. SPP1 has a role in tumor progression [44], in metastasis in BC [45], and in recurrence in ER-positive BC [46]. STAT3 is a transcription factor and regulates various aspects of BC, such as cancer onset, progression, proliferation, metastasis, and chemoresistance [47,48,49,50]. As for HK2, it has been shown to be required for tumor initiation and maintenance [51,52], and HIF-1α has been shown to upregulate HK2 expression, leading to pro-survival and epithelial-to-mesenchymal transition in BC [53,54]. In agreement with our findings, HIF-1α expression has been associated with cancer cell stemness, progression, resistance, and poor prognosis [55,56,57].

Taken together, these data suggest an important role of *TBC1D9* as a modulator of BC migration and tumor growth. Furthermore, the high expression on *TBC1D9* in HER2-positive BC tissue samples and its differential role in these subtypes as compared to luminal A and TNBC could be an interesting avenue for future investigation. Most importantly, low *TBC1D9* expression is indicative of a TNBC phenotype, and its low expression is correlated with high-grade tumors and worse survival outcomes. This study identified a new marker (TBC1D9) that differentiates TNBC from non-TNBC (apart from ER, PR, and HER2), the further investigation of which will be key in identifying a therapeutic target for the TNBC subgroup irrespective of its heterogeneity. The major limitation of our study was the low sample sizes for the luminal A, luminal B, and HER2 subgroups; thus, a follow-up study with larger sample sizes of all BC subtypes, including non-TNBC (luminal A, luminal B, and HER2), would further strengthen our findings.

## 5. Conclusions

In the present study, we identified a novel gene, *TBC1D9*, the low expression of which is indicative of TNBC phenotype, irrespective of the molecular heterogeneity of TNBC. This gene could be used as a potential therapeutic target for TNBC by manipulating either its expression (i.e., increasing expression) or its effector molecules in combination with other available therapies. Furthermore, we identified *TBC1D9* as an important regulator of migration and tumor growth in BC.

## 6. Future Perspectives

1. It would be interesting to understand why *TBC1D9* behaves differently in HER2-positive BC cell lines and how this information could be used in targeting HER2-positive BC.
2. Is the difference in the altered oncogenic signaling upon KD of *TBC1D9* in luminal A and luminal B due to the presence of HER2 in luminal B subtypes?

## Figures and Tables

**Table 1:** Spearman correlations of markers with H-scores for TBC1D9 expression.

	All BCs (*n* = 83)		IDC (*n* = 70)		TNBC (*n* = 58)		Non-TNBC Including DCIS (*n* = 25)		Non-TNBC (*n* = 12)	
Markers	Rho	*p*-Value	Rho	*p*-Value	Rho	*p*-Value	Rho	*p*-Value	Rho	*p*-Value
**Tumor Size (mm)**	**−0.25**	**0.02**	−0.22	0.06	−0.14	0.29	**−0.49**	**0.01**	**−0.80**	**0.01**
**Ki-67 percent staining**	**−0.29**	**0.008**	−0.11	0.34	0.03	0.83	−0.04	0.85	−0.04	0.52
**CD24** **H-score**	0.20	0.07	**0.27**	**0.02**	**0.36**	**0.006**	−0.09	0.66	−0.09	0.85
**Claudin 3** **H-score**	0.08	0.48	0.10	0.41	0.18	0.18	0.25	0.24	0.25	0.53
**Claudin 4** **H-score**	−0.11	0.34	−0.02	0.84	0.04	0.76	−0.15	0.46	−0.15	0.83

Abbreviations: IDC, invasive ductal carcinoma; DCIS, ductal carcinoma in situ; TNBC, triple-negative breast cancer. Significant correlations are highlighted in bold.

## Data Availability

Publicly available datasets were analyzed in this study. The expression data for *TBC1D9* in the BC dataset of the GENT2 and ULCAN databases can be found at http://gent2.appex.kr/gent2/ and http://ualcan.path.uab.edu/, respectively. The data were accessed on 11 January 2021. Survival data for *TBC1D9* can be found at https://kmplot.com/analysis/, which was accessed on 7 June 2021.

## Supplementary Materials

The following are available online at https://www.mdpi.com/article/10.3390/cancers13143557/s1, Figure S1: Representative images of IHC staining for different antibodies, Figure S2: TBC1D9 expression in BC tissue samples from our cohort, Figure S3: Validation of signaling altered by knockdown of TBC1D9 in BC cell lines, Table S1: Molecular classification of BC cell lines, Table S2: Primer sequence.
