# Supplementary material for: TBC1D9: An Important Modulator of Tumorigenesis in Breast Cancer
Source: Cancers (Basel). 2021 Jul 16;13(14):3557. doi: 10.3390/cancers13143557 (PMC8304074; doi:10.3390/cancers13143557)
Supplement: Supplementary file 1 [file cancers-13-03557-s001.zip › cancers-1279785-SI.pdf]

**Table S1.** Molecular classification of BC cell lines.

| Cell Line | ER | PR | HER2 | Subtype |
|---|---|---|---|---|
| MCF7 | + | + | - | Luminal A |
| T47D | + | + | - | Luminal A |
| BT-474 | + | + | + | Luminal B |
| SKBr3 | - | - | + | HER2 |
| JIMT-1 | - | - | + | HER2 |
| SUM159PT | - | - | - | TNBC |
| SUM149PT | - | - | - | TNBC |
| MDA-MB-231 | - | - | - | TNBC |
| MDS-MB-468 | - | - | - | TNBC |
| BT-549 | - | - | - | TNBC |

Abbreviations: +: Positive; -: Negative.

**Table S2.** Primer sequence.

| Gene Symbol | Description | Size (bp) | Primer sequence 5'→3' Forward/Reverse |
|---|---|---|---|
| **TBC1D9** | TBC1 Domain Family Member 9 | 90 | CAGCAGCCTCGTCTCCTCCAG/ GGGACGCTGTTGCCATTTAGG |
| **ARL8A** | ADP ribosylation factor like GTPase 8A | 116 | TAGTCCTGGGTAACAAGCGAGAC / TGCAAGAGATGGAGTAGCAGCAG |
| **ARL8B** | ADP ribosylation factor like GTPase 8B | 122 | TGATACCCACAGTGGGCTTCAAC / TGACTCCTCTGCAATACCGCTC |
| **PLK1** | Polo like kinase 1 | 123 | GCACAGTGTCAATGCCTCCAAGC / GCCGTACTTGTCCGAATAGTCC |
| **HIF-1$\alpha$** | Homo sapiens hypoxia inducible factor 1 subunit alpha (HIF1A), 3 transcripts | 144 | TATGAGCCAGAA-GAACTTTTAGGCC/CCACCTCTTTGG-CAAGCATCCTG |
| **HK2** | Hexokinase 2 | 94 | GAATGGGAAGTGGGGTGGA / TGTGGTCAAAGAGCTCGTCC |
| **SPP1** | Homo sapiens secreted phosphoprotein 1 | 108 | AAGAAGTTTCGCAGACCTGACATC / AACGGGGATGGCCTTGTATGC |
| **STAT3** | Signal transducer and activator of tran-scription 3 | 133 | CCTTTGAGACCGAGGTGTATCACC / GGTCAGCATGTTGTACCACAGG |
| **HPRT1** | Homo sapiens hypoxanthine phosphori-bosyltransferase 1 | 86 | CGTCGTGATTAGTGATGATGAACCA/ ACACCCTTTCCAAATCCTCAGC |
| **GAPDH** | Homo sapiens glyceraldehyde-3-phos-phate dehydrogenase | 93 | CGGGGCTCTCCAGAACATCAT/ ATGCCAGTGAGCTTCCCGTTC |

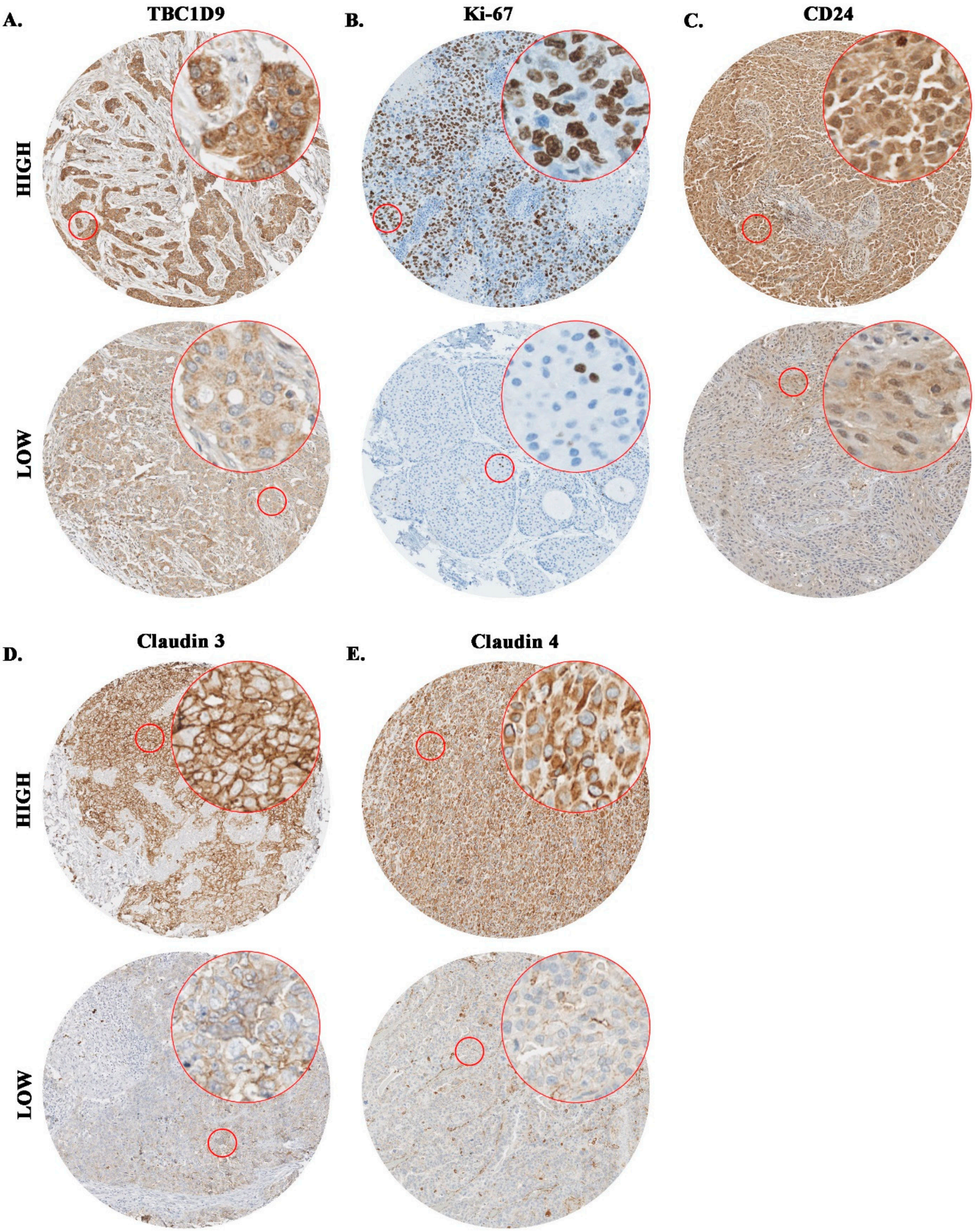


**Figure S1.** Representative images of IHC staining for different antibodies. Figure S1: Representative images of IHC staining for different antibodies. TBC1D9 (**A**), Ki-67 (**B**), CD24 (**C**), Claudin 3 (**D**), and Claudin 4 (**E**). Scale bar 100µm.

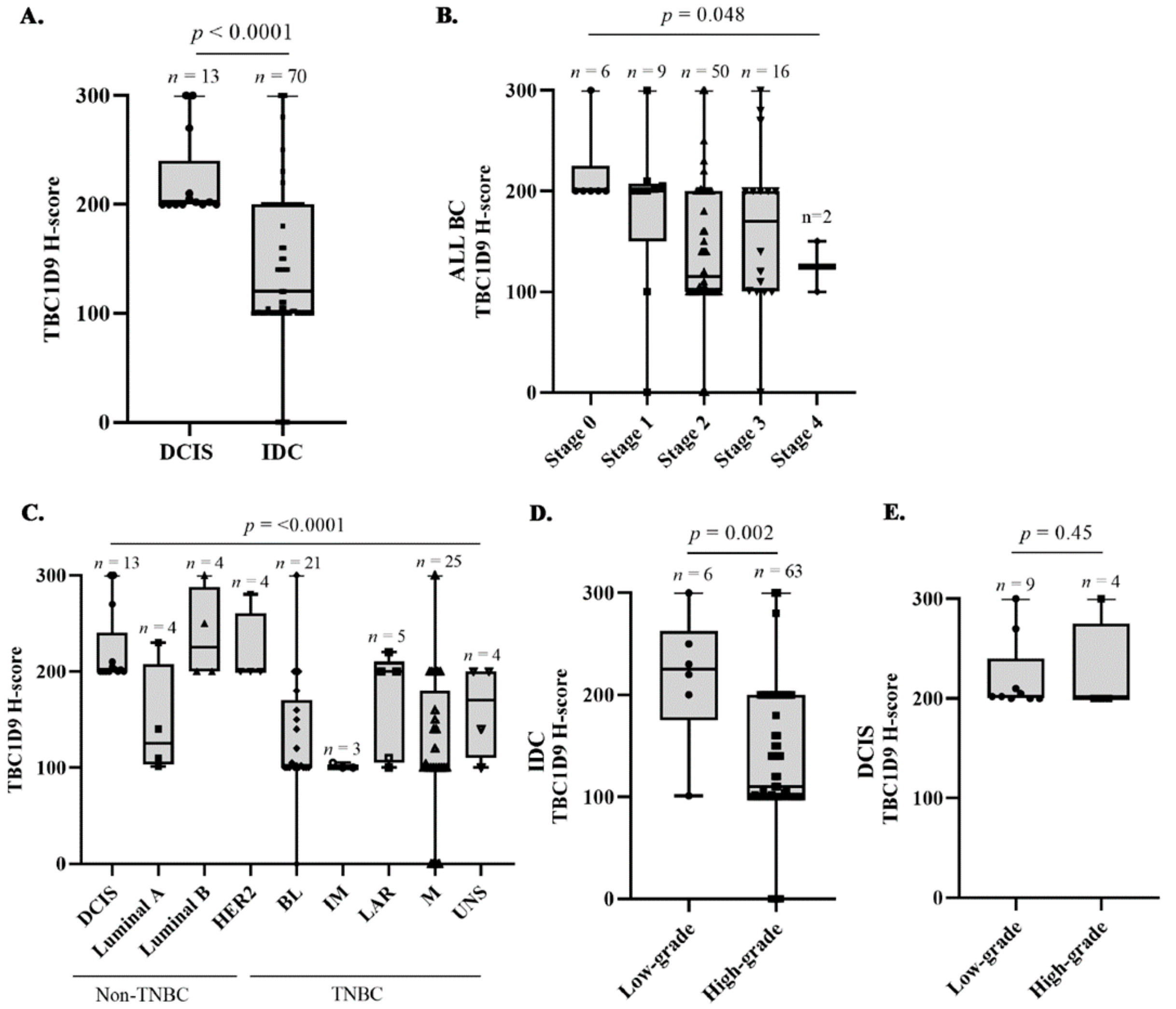


**Figure S2.** TBC1D9 expression in BC tissue samples from our cohort. Stratified by DCIS vs. IDC (**A**) and according to BC stage (**B**) and the molecular subtypes of BC (**C**). BC samples were stratified by grade for IDC (**D**) and DCIS (**E**). BL, = basal-like, IM = immunomodulatory, LAR = luminal androgen receptor, M = mesenchymal, UNS = unclassified.

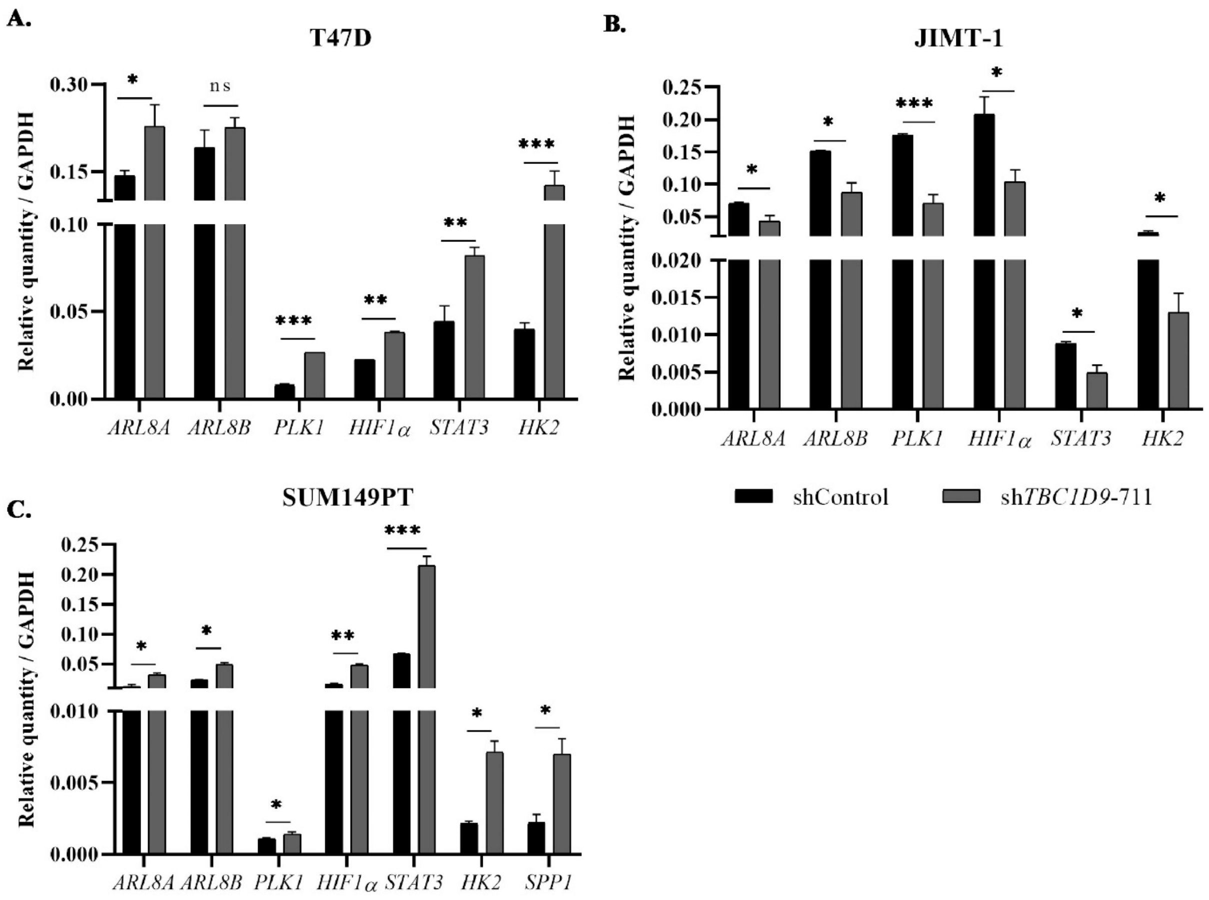


**Figure S3.** Validation of signaling altered by knockdown of TBC1D9 in BC cell lines. T47D: luminal A (**A**), JIMT-1: HER2 (**B**), SUM149PT: TNBC (**C**), and HK = *GAPDH*. ; ns: not significant
